# Correction: Time Adaptation Shows Duration Selectivity in the Human Parietal Cortex

**DOI:** 10.1371/journal.pbio.1002296

**Published:** 2015-11-04

**Authors:** Masamichi J. Hayashi, Thomas Ditye, Tokiko Harada, Maho Hashiguchi, Norihiro Sadato, Synnöve Carlson, Vincent Walsh, Ryota Kanai

Reference 70 is a duplicate of Reference 12. A corrected version of the paper, S1 Manuscript, has been provided below. In this file, the duplicated reference (70) has been removed from the Reference list and all subsequent in-text reference citations have been revised.

## Supporting Information

S1 Manuscript Corrected manuscript file. (DOCX)
