# Supplementary figures and images for: Time Adaptation Shows Duration Selectivity in the Human Parietal Cortex
Source: PLoS Biol. 2015 Sep 17;13(9):e1002262. doi: 10.1371/journal.pbio.1002262 (PMC4574920; doi:10.1371/journal.pbio.1002262)

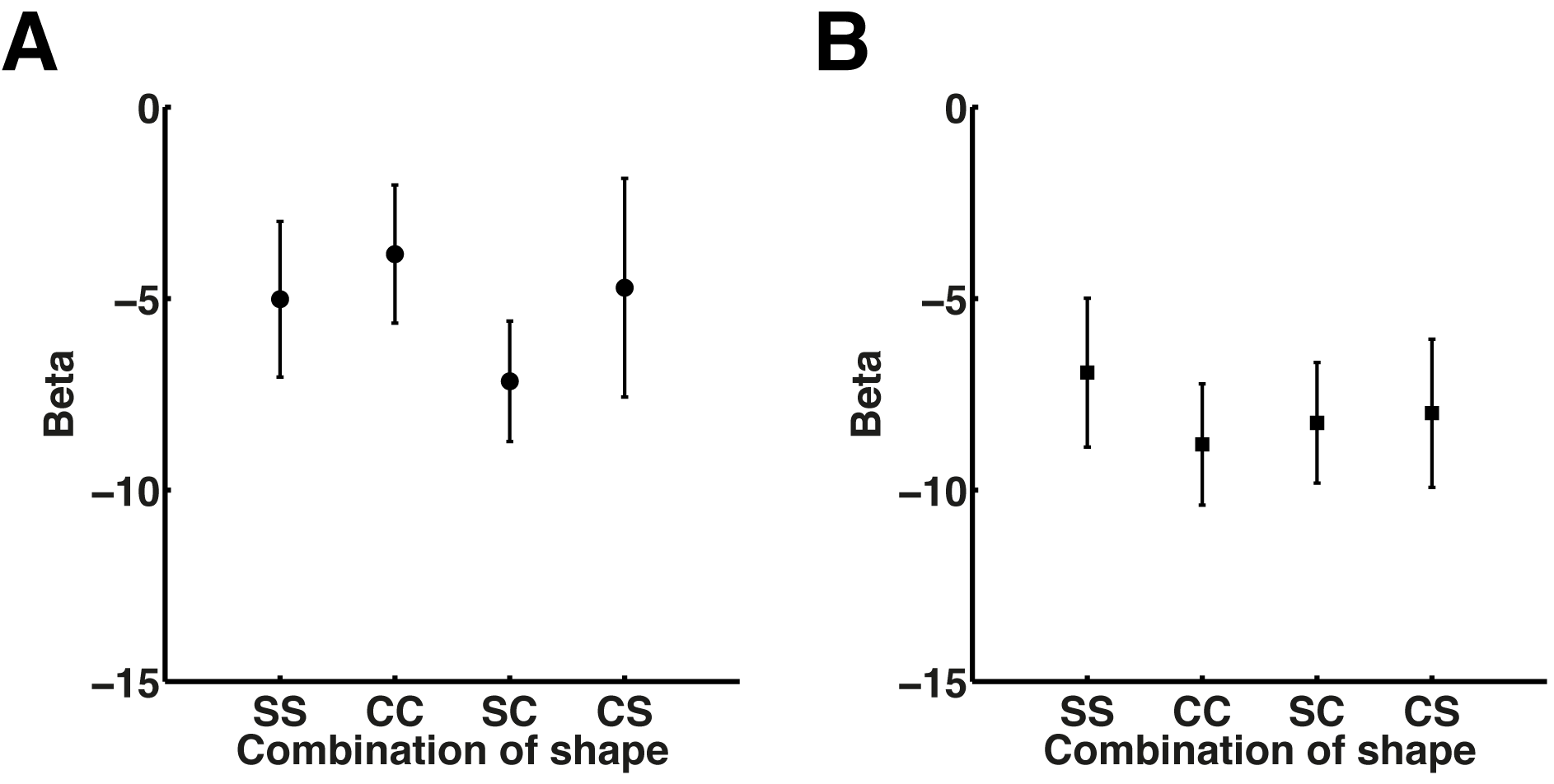

Supplement: S1 Fig — (A) Plots of the beta values under each set of shapes at the peak coordinates of the clusters in the right SMG (x, y, z = 58, −42, 30) that showed duration adaptation effects during the time task in experiment 1 (see Fig 2A). (B) Plots of the beta values under each set of shapes at the peak coordinates of the clusters in the right SMG (x, y, z = 62, −34, 32) that showed a duration adaptation effect during the time task in experiment 2 (see Fig 2C). Letters on the x-axes represent combinations of shapes for reference and test stimuli: square-square (SS), circle-circle (CC), square-circle (SC), and circle-square (CS). Error bars indicate standard errors of the mean. Please refer to S1 Data for the numerical values underlying these figures. (TIF) [file pbio.1002262.s002.tif]

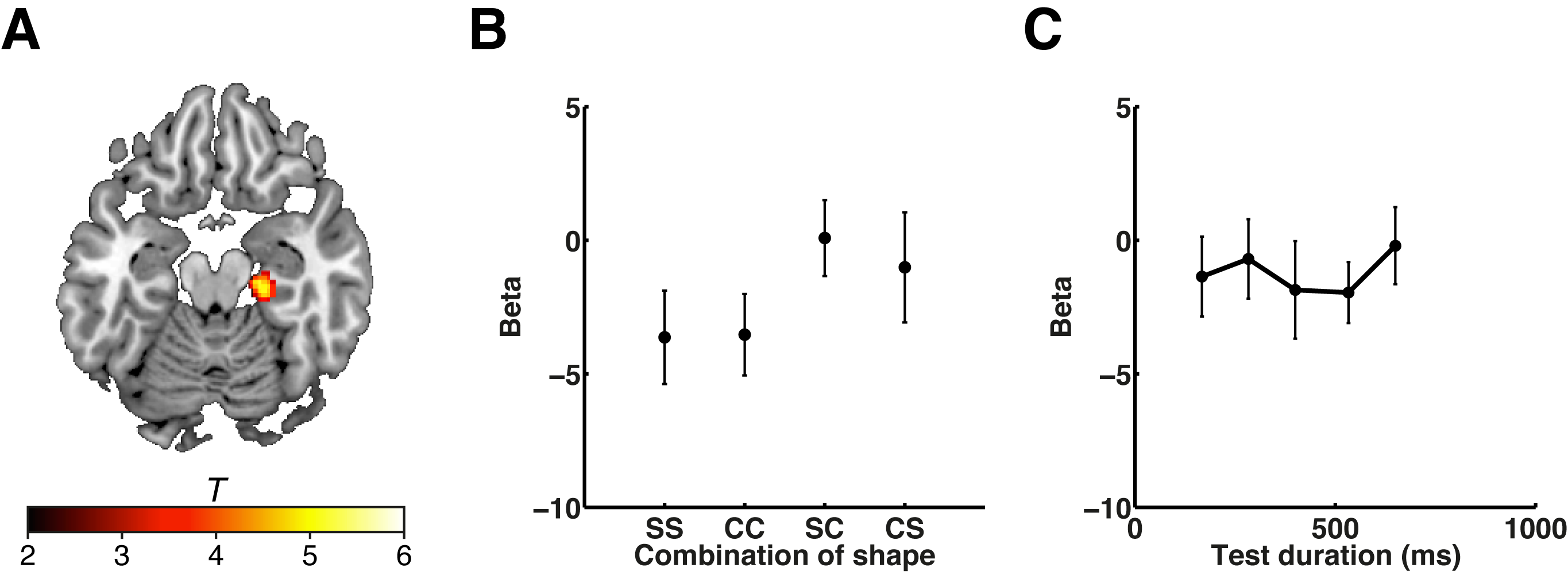

Supplement: S2 Fig — (A) Cluster showing shape adaptation is shown in axial (bottom left, z = −18) slices of the standard brain. Plots of the beta values for each set of shapes during the shape task (B) and for each set of stimulus durations during the time task (C) at the peak coordinates of the cluster in the right parahippocampal gyrus (x, y, z = 20, −22, −18). The right parahippocampal gyrus showed a significant adaptation to the repetition of the same shape but not to the repetition of the same duration. Color scale indicates T-values. Letters on the x-axis represent combinations of shapes for reference and test stimuli: square-square (SS), circle-circle (CC), square-circle (SC), and circle-square (CS). Error bars indicate standard errors of the mean. Please refer to S1 Data for the numerical values underlying (B) and (C). (TIF) [file pbio.1002262.s003.tif]

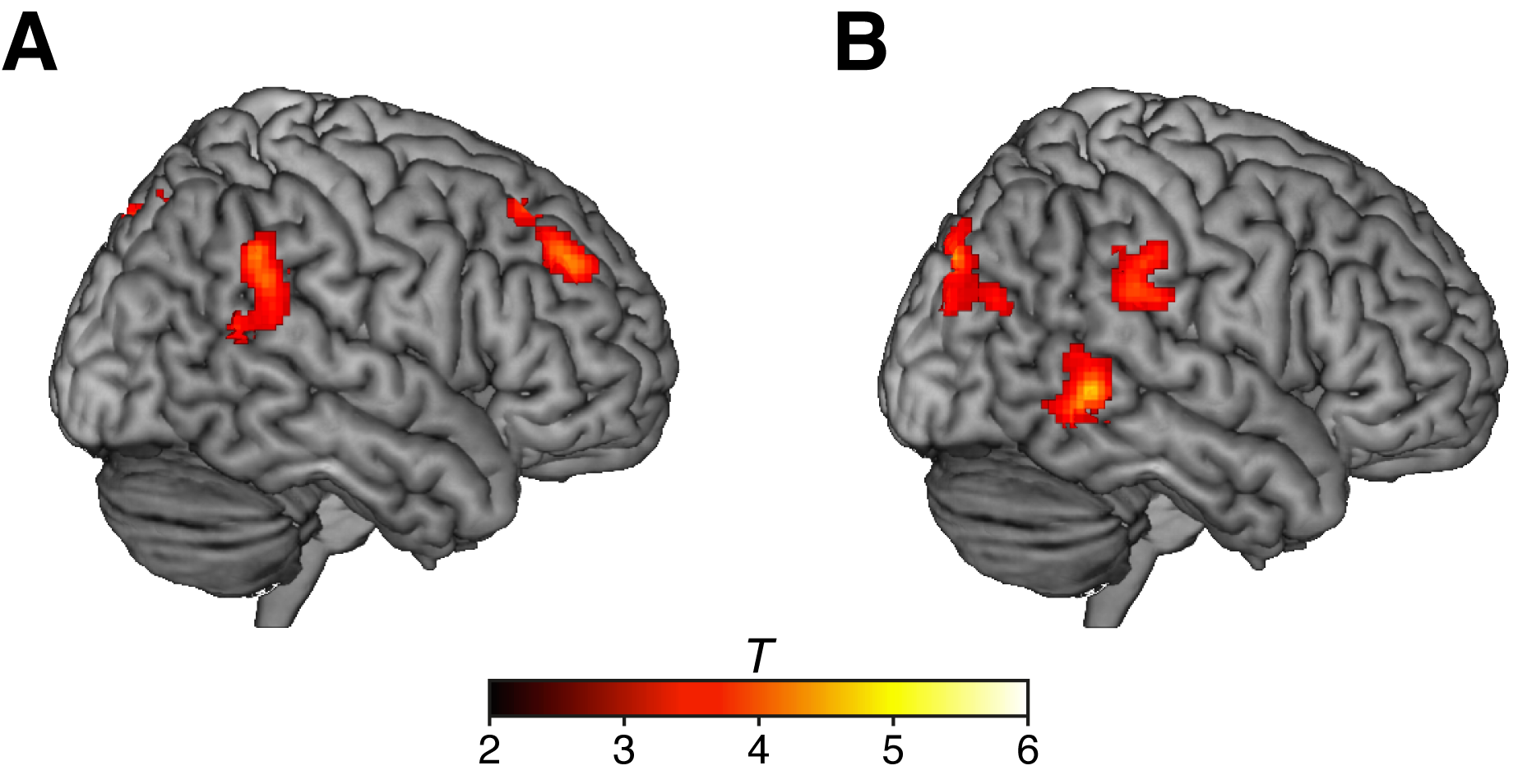

Supplement: S3 Fig — Clusters highlighted by the contrast (duration adaptation during time task > shape adaptation during shape task) are shown for experiments 1 (A) and 2 (B). The color scale indicates the T-values. (TIF) [file pbio.1002262.s004.tif]

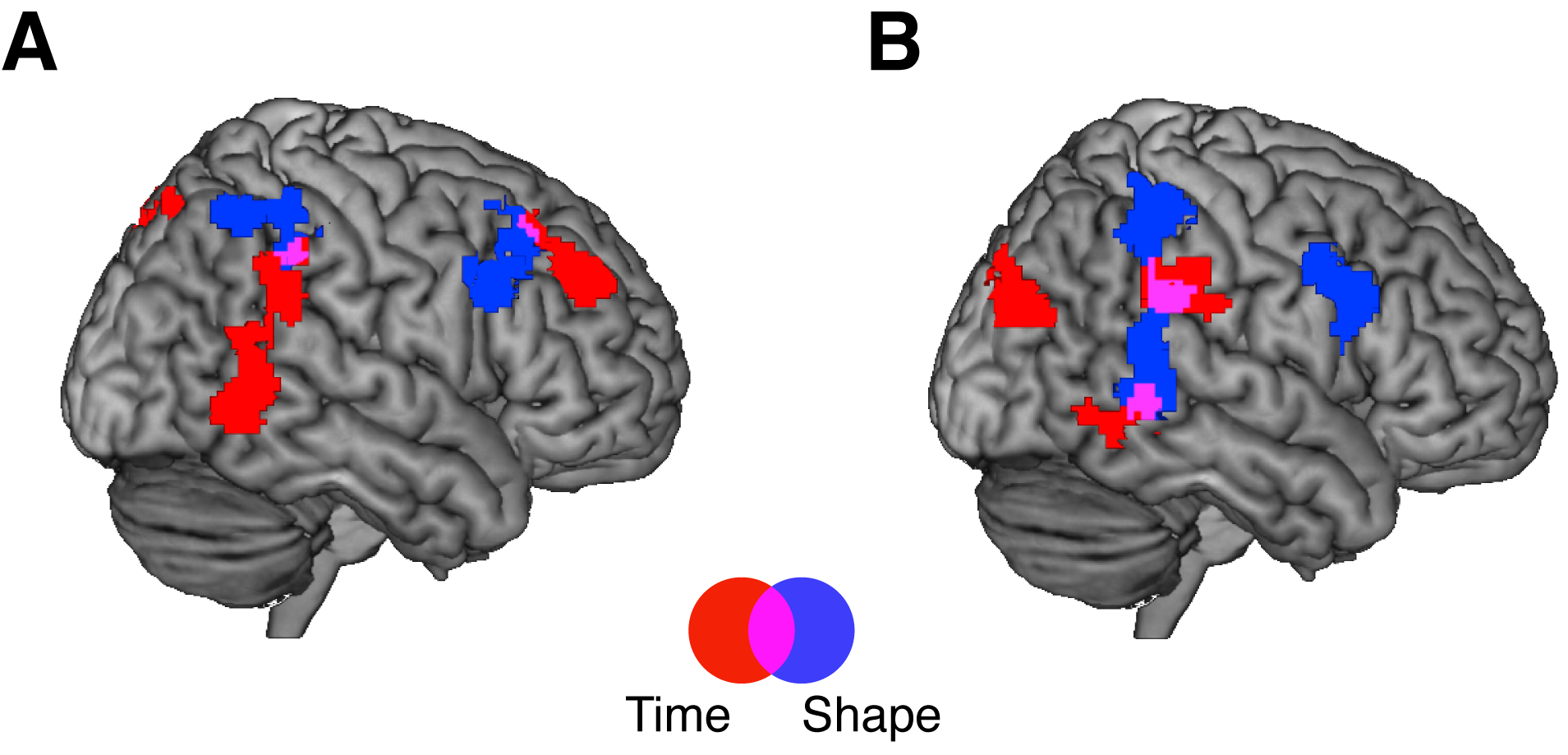

Supplement: S4 Fig — Red clusters represent areas showing duration adaptation effects during the time task (corresponding to the results shown in Fig 2A and 2C), and blue areas represent those showing duration adaptation effects during the shape task (corresponding to the results shown in Fig 3A and 3C) in experiment 1 (A) and experiment 2 (B). Overlapped areas are colored magenta, as shown in the legend. (TIF) [file pbio.1002262.s005.tif]
