# Supplementary material for: Time Adaptation Shows Duration Selectivity in the Human Parietal Cortex
Source: PLoS Biol. 2015 Sep 17;13(9):e1002262. doi: 10.1371/journal.pbio.1002262 (PMC4574920; doi:10.1371/journal.pbio.1002262)
Supplement: S1 Table — (DOC) [file pbio.1002262.s006.doc]

|  |  | MNI coordinates | | |  |  |
| --- | --- | --- | --- | --- | --- | --- |
| Cluster size (mm^3^) | T-value | x | y | z | Side | Location |
| *Effect of time adaptation during time task* | | | | |  |  |
| 9936 | 4.63 | 2 | -64 | 58 | R | Precuneus |
|  | 4.27 | 0 | -46 | 48 | L | Middle cingulate cortex |
|  | 4.22 | 22 | -72 | 50 | R | Superior parietal lobule |
|  | 4.20 | 0 | -52 | 50 | L | Precuneus |
| 6000 | 4.62 | 60 | -58 | -4 | R | Inferior temporal gyrus |
|  | 4.20 | 54 | -44 | -2 | R | Middle temporal gyrus |
|  | 4.18 | 66 | -54 | 18 | R | Superior temporal gyrus |
|  | 3.75 | 58 | -42 | 30 | R | Supramarginal gyrus |
| 3984 | 4.09 | -16 | -10 | 20 | L | Caudate Nucleus |
|  | 4.04 | -18 | 10 | -6 | L | Putamen |
| 3136 | 4.92 | 32 | 42 | 36 | R | Middle frontal gyrus |
| *Time adaptation during time task > Shape adaptation during shape task* | | | | | | |
| 3072 | 4.03 | 2 | -66 | 56 | R | Precuneus |
|  | 3.76 | 0 | -46 | 48 | L | Middle cingulate cortex |
|  | 3.56 | -16 | -76 | 54 | L | Superior parietal lobule |
|  | 3.52 | -10 | -76 | 54 | L | Precuneus |
|  | 3.40 | 20 | -74 | 50 | R | Superior parietal lobule |
| 2576 | 4.24 | 58 | -46 | 36 | R | Supramarginal gyrus |
|  | 3.85 | 66 | -54 | 18 | R | Superior temporal gyrus |
| 1792 | 4.31 | 34 | 42 | 36 | R | Middle frontal gyrus |
| *Effect of time adaptation during shape task* | | | | |  |  |
| 3792 | 4.23 | 54 | -42 | 58 | R | Superior parietal lobule |
|  | 4.22 | 52 | -40 | 40 | R | Supramarginal gyrus |
|  | 3.94 | 52 | -56 | 50 | R | Inferior parietal lobule |
| 2632 | 4.02 | 44 | 26 | 44 | R | Middle frontal gyrus |
|  | 3.90 | 54 | 18 | 34 | R | Inferior frontal gyrus |
|  | 3.45 | 38 | 24 | 54 | R | Middle frontal gyrus |
| 1496 | 3.96 | 8 | -68 | 44 | R | Precuneus |
| L, left; R, right. | | | | | | |
