# Supplementary material for: Time Adaptation Shows Duration Selectivity in the Human Parietal Cortex
Source: PLoS Biol. 2015 Sep 17;13(9):e1002262. doi: 10.1371/journal.pbio.1002262 (PMC4574920; doi:10.1371/journal.pbio.1002262)
Supplement: S2 Table — (DOC) [file pbio.1002262.s007.doc]

|  |  | MNI coordinates | | |  |  |
| --- | --- | --- | --- | --- | --- | --- |
| Cluster size (mm^3^) | T-value | x | y | z | Side | Location |
| *Effect of time adaptation during time task* | | | | |  |  |
| 3720 | 4.45 | 40 | -76 | 24 | R | Middle occipital gyrus |
| 3480 | 4.55 | 62 | -34 | 32 | R | Supramarginal gyrus |
| 3264 | 4.61 | 56 | -46 | -8 | R | Middle temporal gyrus |
|  | 3.44 | 56 | -60 | -4 | R | Inferior temporal gyrus |
| *Time adaptation during time task > Shape adaptation during shape task* | | | | | | |
| 3736 | 4.76 | 60 | -44 | -2 | R | Middle temporal gyrus |
|  | 4.57 | 54 | -44 | -10 | R | Inferior temporal gyrus |
| 2296 | 3.94 | 60 | -32 | 28 | R | Supramarginal gyrus |
| 2272 | 3.60 | 50 | -72 | 24 | R | Middle occipital gyrus |
| *Effect of time adaptation during shape task* | | | | |  |  |
| 8728 | 4.72 | 48 | -42 | 44 | R | Supramarginal gyrus |
|  | 4.68 | 50 | -42 | 48 | R | Inferior parietal lobule |
|  | 4.59 | 68 | -42 | 2 | R | Middle temporal gyrus |
|  | 4.48 | 54 | -40 | 58 | R | Superior parietal lobule |
|  | 4.10 | 58 | -44 | 18 | R | Superior temporal gyrus |
| 6848 | 5.89 | -58 | 14 | 30 | L | Inferior frontal gyrus |
|  | 3.69 | -48 | -4 | 40 | L | Precentral gyrus |
|  | 3.46 | -52 | 28 | 32 | L | Middle frontal gyrus |
| 4968 | 4.28 | -58 | -48 | 26 | L | Supramarginal gyrus |
|  | 4.12 | -46 | -46 | 48 | L | Inferior parietal lobule |
| 4600 | 4.06 | 36 | 2 | 30 | R | Precentral gyrus |
|  | 4.04 | 34 | 6 | 28 | R | Inferior frontal gyrus |
| L, left; R, right. | | | | | | |
