# Supplementary material for: Correction: Time Adaptation Shows Duration Selectivity in the Human Parietal Cortex
Source: PLoS Biol. 2015 Nov 4;13(11):e1002296. doi: 10.1371/journal.pbio.1002296 (PMC4633153; doi:10.1371/journal.pbio.1002296)
Supplement: S1 Manuscript — (DOCX) [file pbio.1002296.s001.docx]

**Time Adaptation Shows Duration Selectivity in the Human Parietal Cortex**

Masamichi J. Hayashi^1,2,3,4,5^*, Thomas Ditye^3^, Tokiko Harada^6^, Maho Hashiguchi^6,7^, Norihiro Sadato^6,7,8^, Synnöve Carlson^4,5^, Vincent Walsh^3^, Ryota Kanai^2,3,9^

1 Graduate School of Frontier Biosciences, Osaka University, Suita, Japan,

2 School of Psychology, University of Sussex, Brighton, United Kingdom,

3 Institute of Cognitive Neuroscience, University College London, London, United Kingdom,

4 Brain Research Unit, Department of Neuroscience and Biomedical Engineering, Aalto University School of Science, Espoo, Finland,

5 Department of Physiology, Faculty of Medicine, University of Helsinki, Helsinki, Finland,

6 Division of Cerebral Integration, National Institute for Physiological Sciences, Okazaki, Japan,

7 Department of Physiological Sciences, The Graduate University for Advanced Studies, Okazaki, Japan,

8 Biomedical Imaging Research Center, University of Fukui, Fukui, Japan,

9 Department of Neuroinformatics, Araya Brain Imaging, Tokyo, Japan

* mjhgml@gmail.com

**Abbreviations:** 3-D, three-dimensional; BOLD signal, blood-oxygenation-level-dependent signal; CNV, contingent negative variation; EEG, electroencephalography; fMRI, functional magnetic resonance imaging; GLM, general linear model; GM, gray matter; IPL, inferior parietal lobule; ISI, interstimulus interval; ITG, inferior temporal gyrus; MASCOI, Masked Contrast Images; MNI, Montreal Neurological Institute; MPRAGE, magnetization-prepared rapid acquisition gradient-echo; MTG, middle temporal gyrus; PTC, posterior temporal cortex; SD, standard deviation; SMA, supplementary motor area; SMG, supramarginal gyrus; TMS, transcranial magnetic stimulation; ToM, theory of mind; TPJ, temporoparietal junction; TPJa, anterior TPJ; TPJp, posterior TPJ; VBM, voxel-based morphometry**Abstract**

Although psychological and computational models of time estimation have postulated the existence of neural representations tuned for specific durations, empirical evidence of this notion has been lacking. Here, using a functional magnetic resonance imaging (fMRI) adaptation paradigm, we show that the inferior parietal lobule (IPL) (corresponding to the supramarginal gyrus) exhibited reduction in neural activity due to adaptation when a visual stimulus of the same duration was repeatedly presented. Adaptation was strongest when stimuli of identical durations were repeated, and it gradually decreased as the difference between the reference and test durations increased. This tuning property generalized across a broad range of durations, indicating the presence of general time-representation mechanisms in the IPL. Furthermore, adaptation was observed irrespective of the subject’s attention to time. Repetition of a nontemporal aspect of the stimulus (i.e., shape) did not produce neural adaptation in the IPL. These results provide neural evidence for duration-tuned representations in the human brain.

**Author Summary**

The human brain has the ability to estimate the passage of time, which allows us to perform complex cognitive tasks such as playing music, dancing, and understanding speech. Scientists have just begun to understand which brain areas become active when we estimate time. However, it still remains a mystery how exactly the information about time is represented in the brain. In this study, we hypothesized that time might be represented by neurons that are specifically tuned to a specific duration, as has been known for simple visual features such as the orientation and the motion direction in the visual cortex. To test this idea, we performed multiple functional magnetic resonance imaging (fMRI) adaptation experiments in which we sought evidence of neuronal adaptation, that is, a reduction in the responsiveness of neurons to repeated presentations of similar durations. Our experiments revealed that the level of brain activity in the right inferior parietal lobule (IPL) was strongly reduced when a stimulus of the same duration was repeatedly presented. This finding was reproduced for a range of subsecond durations. Our results indicate that neurons in the human IPL are tuned to specific preferred durations.

**Introduction**

Time is a fundamental property of our perception and action. Precise time-interval estimation in the range of hundreds of milliseconds is important for motor control, motion detection, and speech recognition and generation, as well as for many other complex sensory motor tasks such as playing music or dancing [[1](#_ENREF_1),[2](#_ENREF_2)]. Previous studies of the neural representation of stimulus duration have found various forms of gradual, time-dependent changes in neural activity. For example, the temporal probability of the occurrence of upcoming events, known as the “hazard rate,” modulates the neural firing rate in the lateral intraparietal region in monkeys [[3](#_ENREF_3)]. Human neuroimaging studies demonstrated that such temporal modulation of firing rate during an anticipation period is reflected in a time-varying increase or decrease of the blood-oxygenation-level-dependent (BOLD) signal in the primary visual cortex, right supramarginal gyrus (SMG), supplementary motor area (SMA), right middle frontal cortex, and cerebellar vermis in humans [[4](#_ENREF_4),[5](#_ENREF_5)]. It has also been reported that elapsed time is represented by the time-dependent ramping activity of neurons in the posterior parietal cortex in monkeys [[6](#_ENREF_6)]. In humans, similar time-dependent increases of BOLD responses during encoding of long time intervals (9 and 18 s) have been reported in the posterior insula and superior temporal cortex [[7](#_ENREF_7)]. Although duration information is indubitably present in such time-dependent changes in neural activity, it is not known whether durations are explicitly coded in the human brain—in other words, whether there are tuning properties for specific durations.

Theoretical models such as the striatal beat-frequency model [[8](#_ENREF_8)], the labeled-line model [[9](#_ENREF_9)], and the population clock model [[10](#_ENREF_10)] have predicted that elapsed time is explicitly represented by the selective firing of a population of neurons in response to the stimulus durations to which they are tuned [[11](#_ENREF_11)]. There is currently some behavioral evidence for the notion of such channel-based processing of time intervals [[12](#_ENREF_12),[13](#_ENREF_13)]. However, potential neural substrates with duration-tuned response properties have not yet been documented.

Here, by using functional magnetic resonance imaging (fMRI) adaptation, we aimed to identify brain regions that show explicit representations of stimulus duration. fMRI adaptation is based on the principle that repetition of an identical stimulus feature produces an immediate decrease in the BOLD signal by repetitive activation of the same subpopulation of neurons [[14](#_ENREF_14),[15](#_ENREF_15)]. If a brain area contains neural populations that are sensitive to the repeated stimulus feature, the BOLD signal shows graded adaptation depending on the perceptual similarity in the stimulus feature space between consecutive presentations [[16](#_ENREF_16),[17](#_ENREF_17)]. A large number of previous studies have shown that perception of time obeys Weber’s Law where the perceptual discriminability of two intervals depends on the ratio of their physical differences (deviation ratio) [[18–22](#_ENREF_18)], but not on the absolute differences. The deviation ratio has been used as a proxy for perceptual discriminability in adaptation paradigms elsewhere [[17](#_ENREF_17)]. According to the population clock model that assumes explicit time representations, duration-tuned neurons are assumed to fire selectively when a time-specific neural firing pattern is submitted from another population of neurons [[10](#_ENREF_10)]. We hypothesized that, if there exist duration-tuned neurons, they would be selectively activated by stimulus offset and show weaker BOLD responses when similar durations are repeated.

We used fMRI adaptation and a broad range of stimulus durations to identify brain regions that showed adaptation to two consecutive visual stimuli of the same duration or slightly different durations. First, we identified brain areas that showed fMRI adaptation for 400 ms of stimulus duration. In the second experiment, we examined whether the brain areas that were identified in the first experiment also showed adaptation to other stimulus durations by replicating the same experiment with a slightly longer stimulus duration (i.e., 600 ms). With these two experiments, we aimed to establish generality and robustness of fMRI adaptation to specific durations. Furthermore, we asked whether such adaptation would require explicit performance of a duration estimation task or would be observed independently of the task relevance of duration estimation. For this purpose, we examined neural adaptation during both an explicit duration discrimination task and during an implicit, nontemporal shape discrimination task. Finally, in a control experiment, we confirmed that the neural adaptation that we observed in the above experiments was specific to time and was not observed in the case of repetition of a nontemporal stimulus feature (i.e., shape).

**Results**

**Experiments 1 and 2: fMRI Adaptation to Stimulus Durations of 400 and 600 ms**

Two groups of participants performed a duration discrimination task and a shape discrimination task as instructed at the beginning of each block (Fig 1A). In the tasks, participants made same-or-different judgments on two successively presented stimuli, but on different aspects of the stimuli (i.e., duration or shape). In each trial, a reference stimulus (adaptor) was presented for a fixed duration, followed by a test stimulus presented for a variable duration. In experiment 1, the duration of the reference stimulus was 400 ms and that of the test stimulus was 167, 283, 400, 533, or 650 ms, whereas in experiment 2 the duration of the reference stimulus was 600 ms and that of the test stimulus was 250, 433, 600, 783, or 967 ms. The shape of the reference and test stimuli was either a square or a circle (i.e., the sequence presented was square-square [SS], circle-circle [CC], square-circle [SC], or circle-square [CS]). To minimize the effect of response preparation and selection processes on the offset of the test stimulus, the key correspondences were randomized on a trial-by-trial basis and were indicated by a response cue presented after a variable interval of 1.0–3.0 s following the offset of the test stimulus. Data of 12 (experiment 1) and 14 (experiment 2) participants were analyzed.

**Fig 1. Stimulus sequence, mean task performances, and deviation ratios in experiments 1 and 2.** (A) The stimulus sequence. Participants were asked to respond with whether the durations of two sequentially presented visual stimuli were the same or different in the time task and whether the shapes of the two stimuli were the same or different in the shape task. (B) Mean task performances in experiments 1 (blue) and 2 (red). Proportions of the “same” responses for the time task were fitted by Gaussian functions for presentation purpose (left). The proportion of the “same” response for the shape task is shown for each combination of reference and test shapes (right). Letters on the *x*-axes represent combinations of shapes for reference and test stimuli: square-square (SS), circle-circle (CC), square-circle (SC), and circle-square (CS). Please refer to S1 Data for the numerical values underlying these figures. (C) Plots of deviation ratios for each set of stimulus durations in experiments 1 (blue) and 2 (red).

**Behavior**

The proportions of “same” responses in the time task were fitted by a Gaussian function for individuals’ behavioral data. Gaussian functions were also fitted to the task performance means for presentation purpose (Fig 1B). The centers of the fitted Gaussian curves did not significantly deviate from the physical durations, showing that perceived durations were not biased from physical stimulus durations in experiment 1 (center = 399.3 ± 32.8, *t*_11_ = −0.070, *p* = 0.946; standard deviation [SD] = 130.0 ± 27.1) and experiment 2 (center = 579.7 ± 51.7, *t*_13_ = −1.465, *p* = 0.167; SD = 162.2 ± 28.3).

**Neural Adaptation to Time during the Time Task**

To identify which brain regions showed graded adaptation to the duration of the reference stimulus (400 or 600 ms), we analyzed the offset response to a test stimulus of variable duration. We hypothesized that BOLD responses to the test stimulus would recover from adaptation according to the deviation ratio between the reference and test stimulus durations (i.e., [longer duration − shorter duration] / shorter duration) (Fig 1C). This analysis revealed that a number of brain areas showed the expected pattern of a neural adaptation effect, primarily in the right hemisphere (Fig 2A and S1 Table for experiment 1 and Fig 2C and S2 Table for experiment 2). Importantly, neural adaptation to both the 400-ms (experiment 1) and the 600-ms (experiment 2) reference duration was found in the right inferior parietal lobule (IPL), corresponding to the SMG, and in the posterior temporal cortex (PTC), including the middle and inferior temporal gyri (MTG/ITG). Plots of the effects at the peak coordinates of the right SMG are shown in Fig 2B (experiment 1) and 2D (experiment 2). Note that, however, values of the regression coefficient (i.e., beta) in most of the test durations were negative, indicating that the overall right SMG activity decreased when the test stimulus had terminated and that the adaptation effect was reflected in the degree of reduction in the BOLD signal. We interpreted that this pattern of BOLD response was generated by mixed effects of general task-related suppression and duration-selective activations within a relatively small neural population. (See Discussion for further details.)

**Fig 2. Duration adaptation effects during the time task in experiments 1 and 2.** (A) Results of experiment 1. Clusters showing duration adaptation effects during the time task are shown on a standard brain. (B) Plots of the beta values at the peak coordinates of the cluster in the right SMG (*x*, *y*, *z* = 58, −42, 30) for each set of stimulus durations. (C) Results of experiment 2. Clusters showing duration adaptation effects during the time task are shown on a standard brain. (D) Plots of the beta values at the peak coordinates of the cluster in the right SMG (*x*, *y*, *z* = 62, −34, 32) for each set of stimulus durations. The color scales indicate the *T*-values. Error bars indicate standard errors of the mean. Please refer to S1 Data for the numerical values underlying (B) and (D).

**No Adaptation to Shape Repetition in IPL and PTC**

One possible explanation for the greater response of the right IPL and PTC to “different” conditions compared with “same” conditions is that the responses reflect general same-or-different decisions and are thus not a reflection of neural adaptation to repeated stimuli of the same duration. To address this possibility, we next examined whether adaptation occurred in response to repetition of the same shape during the shape task that shared processes of same-or-different decisions with the time task.

The results showed that neither the right IPL nor the PTC was sensitive to repetitions of the same shape during the shape task in either experiment 1 or experiment 2. Plots of the beta values in the right IPL are shown in S1 Fig (for experiments 1 [S1A Fig] and experiment 2 [S1B Fig]). Instead, we found a significant effect of shape repetition in the right parahippocampal gyrus, but only in experiment 1 (see S2 Fig for further details). A direct comparison between duration adaptation in the time task and shape adaptation in the shape task confirmed that the right IPL and PTC adapted specifically to repetitions of the same duration, but not to repetitions of the same shape (S3A Fig and S1 Table for experiment 1, S3B Fig and S2 Table for experiment 2). These results suggested that neural adaptation in these regions was specifically linked to repetition of a stimulus duration rather than to decision processes regarding same versus different.

Although we modeled the offset responses for the test stimuli to make the design matrix comparable to that for time, shape adaptation may be observed at the onset of the test stimulus rather than offset, because unlike duration, the information about shape is already available at the stimulus onset. Thus, setting regressors at the onsets of the test stimuli might be considered more appropriate. To address this issue, we analyzed the data of experiments 1 and 2 by setting regressors at the onsets of the test stimuli instead of at their offsets. The results of this analysis were essentially the same as the original findings—no significant clusters were found for experiments 1 and 2, but clusters in the right parahippocampal gyrus and hippocampus were significant in experiment 1, only at slightly more liberal threshold (*p* < 0.001 voxel-level uncorrected, cluster size > 100 voxels). These results further confirm our interpretation that the neural adaptation in the right IPL and PTC are specifically associated with the repetition of the identical stimulus duration.

**Duration Adaptation Occurs Regardless of the Task Relevance of Duration**

Adaptation to duration occurred not only when the participants were performing the time task but also when they were engaged in the shape task, in which duration information was not explicitly estimated. We analyzed the degree of adaptation to repetition of duration, as above, under conditions in which the participants were engaged in the shape task. In both experiment 1 and experiment 2, neural adaptation to time during the shape task was observed in the right IPL (including the SMG) (Fig 3A and 3B and S1 Table for experiment 1, Fig 3C and 3D and S2 Table for experiment 2). The clusters in the right IPL (SMG) overlapped with the clusters found in the explicit time estimation task (S4A Fig for experiment 1, S4B Fig for experiment 2), indicating that neural adaptation to duration in the right IPL (SMG) occurred regardless of whether or not duration was explicitly estimated.

**Fig 3. Duration adaptation effects during the shape task in experiments 1 and 2.** (A) Results of experiment 1. Clusters showing duration adaptation effects during the shape task are shown on a standard brain. (B) Plots of the beta values at the peak coordinates of the cluster in the right SMG (*x*, *y*, *z* = 52, −40, 40) for each set of stimulus durations. (C) Results of experiment 2. Clusters showing duration adaptation effects during the shape task are shown on a standard brain. (D) Plots of the beta values at the peak coordinates of the cluster in the right SMG (*x*, *y*, *z* = 48, −42, 44) for each set of stimulus durations. The color scales indicate the *T*-values. Error bars indicate standard errors of the mean. Please refer to S1 Data for the numerical values underlying (B) and (D).

**Experiment 3: Graded Shape Discrimination Task**

One potential concern in experiments 1 and 2 was the possibility that the lack of adaptation to repetition of a shape in the right IPL and PTC could be attributed to the fact that the shapes were simply either identical or clearly different, whereas the duration variation involved finer steps across five different levels. Therefore, differences in the task design in the shape task and the level of difficulty might have contributed to our positive finding only in the time task, but not in the shape task.

To make the task design and the level of difficulty for the shape task comparable to those for the time task in experiments 1 and 2, we varied the degree of shape change in a graded manner in a new control experiment (experiment 3). The oval-shaped test stimuli were varied in width on five levels (i.e., very narrow [VN], narrow [N], same [S], wide [W], and very wide [VW]), whereas the oval-shaped reference stimulus remained the same throughout the experiment (Fig 4A). In this experiment, participants performed only the shape task because the goal of this experiment was to examine whether the right IPL and PTC show adaptation to the repetition of the same shape when the stimulus shape was manipulated at a finer step of five different levels. For this purpose, we did not intermix the time task in this control experiment, as our interest was not to re-examine the effect of duration adaptation established in experiments 1 and 2. Furthermore, we did not expect that the intermixing of the two tasks was essential for this control experiment, because the shape task was also performed in experiments 1 and 2 in separate blocks. Data from 13 participants were analyzed for this graded-shape discrimination task.

**Fig 4. Stimulus sequence, mean task performances, and deviation ratios in experiment 3.** (A) The stimulus sequence. Participants were asked to respond with whether the widths of two sequentially presented visual stimuli (ovals) were the same or different. (B) Mean task performances. The proportion of “same” responses was fitted by a Gaussian function for presentation purpose. Please refer to S1 Data for the numerical values underlying this figure. (C) Plots of deviation ratios for each set of visual stimulus widths.

**Behavior**

The proportions of the “same” response in the shape task were fitted by a Gaussian function for individuals’ behavioral data. Gaussian functions were also fitted to task performance means for presentation purpose (Fig 4B). The center of the fitted Gaussian curveshowed that the perceived stimulus width was estimated as slightly narrower than the physical stimulus width (center = 58.7 ± 0.5, *t*_12_ = −9.236, *p* < 0.001; SD = 4.2 ± 1.2). One-way ANOVA showed that the task difficulty of this experiment was similar to that of the time task in experiments 1 and 2 (*F*_2, 36_ = 0.779, *p* = 0.467), suggesting that any differences in fMRI results across these experiments (i.e., the time task in experiments 1 and 2 and the graded-shape task in experiment 3) were unlikely to be due to differences in task difficulty.

**Right IPL and PTC Do Not Adapt to Shape Repetition**

We expected that, if the adaptation effect found in the right IPL and PTC in experiments 1 and 2 reflected processes of general decision-making regarding same-or-different judgments, these regions would also show a graded adaptation to other stimulus features (i.e., the width of an oval) (Fig 4C). We found that, despite the graded change of the shape in this experiment, the right IPL (SMG) and PTC did not show adaptation to repetition of the same shape. As we did for experiments 1 and 2, setting regressors at the onsets instead of the offsets of the test stimuli did not change the results. Plots of the relationship between BOLD responses and the graded shape conditions in the right SMG (at the same coordinates as in the plots in Fig 2B and 2D) are shown in Fig 5A and 5B. Importantly, the BOLD signal extracted from the right SMG (Fig 5) showed negative regression coefficients, indicating that the right SMG showed reduction of BOLD signal due to attention focus regardless of the task (i.e., duration or shape discrimination task). These results corroborate our conclusion that neural adaptation in the right IPL (SMG) was specific to time and did not reflect general decision-making processes regarding same-or-different judgments.

**Fig 5. Plots of the beta values in the SMG.** Plots of the relationship between beta values and the test stimulus width in experiment 3 are shown at the peak coordinates in the right SMG (*x*, *y*, *z* = 58, −42, 30) that showed a duration adaptation effect in experiment 1 (A) and in the right SMG (*x*, *y*, *z* = 62, −34, 32) that showed a duration adaptation effect in experiment 2 (see Fig 2) (B). Plots of the beta values for each set of test durations at the same coordinates as in Fig 5A and Fig 5B are shown in (C) and (D), respectively. Please note that, while the beta values in (C) showed significant duration adaptation effect, the beta values in (D) (*x*, *y*, *z* = 62, −34, 32) did not reach statistical significance. Error bars indicate standard errors of the mean. Please refer to S1 Data for the numerical values underlying these figures.

Finally, we also examined whether the repetition of the same duration during the graded-shape task showed adaptation in the right IPL. Plots of the relationship between BOLD responses and the test duration conditions at the same coordinates as in Fig 5A and 5B are shown in Fig 5C and 5D. Although the duration adaptation was not very clear at these coordinates, we instead found that the slightly more posterior part of the right IPL (SMG) showed a significant graded adaptation to stimulus durations (Fig 6). These results further support our findings in experiments 1 and 2 that neural adaptation to the repetition of identical duration in the right IPL (SMG) occurred regardless of whether the durations were explicitly estimated.

**Fig 6. Effects of duration adaptation during the graded-shape task in experiment 3.** (A) Results of experiment 3. The right SMG cluster showing duration adaptation effects during the graded-shape task is shown on a standard brain. Plots of the beta values at the peak coordinates of the cluster in the right SMG (*x*, *y*, *z* = 60, −44, 30) for each set of stimulus durations (B) and for each set of stimulus widths (C). The color scale indicates the *T*-values. Error bars indicate standard errors of the mean. Please refer to S1 Data for the numerical values underlying (B) and (C).

**Experiment 4: fMRI Adaptation to Stimulus Durations of 300 and 450 ms**

Having established the specificity of neural adaptation to stimulus duration in the right IPL, we further addressed some other potential concerns. One could argue that the duration adaptation effect observed in experiments 1 and 2 might reflect neural adaptation to the fixed interstimulus interval (ISI) between the reference and test stimuli, but not to the duration of the reference stimulus, because the ISI (0.5 s) was close to the duration of the “same” conditions in these experiments (400 and 600 ms in experiments 1 and 2, respectively). To address this concern, we performed another control experiment (experiment 4) with a longer ISI that varied between 1 s and 2.5 s (0.5-s steps).

Another concern was that participants’ responses were biased toward “same” when a slightly shorter or longer duration was presented as the test stimulus, resulting in considerably lower performances under these conditions. To resolve this issue in experiment 4, we also changed the number of response alternatives and the number of variations in the duration of the test stimuli. Whereas judgments of the test stimulus as “shorter” or “longer” were both classed as “different” in experiments 1 and 2, in experiment 4 these judgments were classed individually as “shorter” or “longer” (Fig 7A, see Materials and Methods for full details). The duration of the reference stimulus was either 300 ms or 450 ms; that of the test stimulus was 200 ms, 300 ms, or 450 ms for the block with a reference duration of 300 ms, and 300 ms, 450 ms, or 667 ms for the block with a reference duration of 450 ms. A response cue was presented after a variable interval of 2.0–3.5 s following the offset of the test stimuli. Data from 20 participants were analyzed in this experiment.

**Fig 7. Stimulus sequence, mean task performances, and deviation ratios in experiment 4.** (A) The stimulus sequence. Participants were asked to respond with whether the duration of the second (test) stimulus was shorter, longer, or the same compared with the first (reference) stimulus. (B) Mean task performances. Proportions of the “same” responses for the 300 ms (blue) and the 450 ms (red) reference blocks were fitted by Gaussian functions for presentation purpose. Please refer to S1 Data for the numerical values underlying this figure. (C) Plots of deviation ratios for each set of stimulus durations in the 300 ms reference block (blue) and in the 450 ms reference block (red).

**Behavior**

Proportions of the “same” responses were fitted by a Gaussian function for individuals’ behavioral data (Fig 7B). Statistical analyses showed that time estimation for the 300 ms block was perceived as slightly longer (centre = 325.5 ± 52.3, *t*_19_ = 2.182; *p* = 0.042; SD = 99.6 ± 60.2), whereas that for the 450 ms block was not biased (centre = 463.4 ± 43.6, *t*_19_ = 1.380, *p* = 0.184; SD = 134.3 ± 30.9). In contrast to the time task in experiments 1 and 2, participants showed comparable accuracy across conditions (*F*_1.88, 35.77_ = 0.610, *p* = 0.540).

**Replication of Duration Adaptation in the Right IPL**

As in experiments 1 and 2, we predicted that the right IPL and PTC would show adaptation to repeated stimuli of the same duration. Our analysis showed a substantial duration adaptation effect in the right IPL (SMG) (Fig 8), whereas the right PTC did not show such significant adaptation. The result in the right IPL is consistent with the findings in experiments 1 and 2 and supports our observation of duration-specific adaptation in the IPL.

**Fig 8. Duration adaptation effects in experiment 4.** (A) The cluster showing a duration adaptation effect is shown on a standard brain. (B) Plots of the beta values under each set of stimulus conditions in the 300ms (blue) and the 450ms (red) reference blocks at the peak coordinates of the cluster in the right SMG (*x*, *y*, *z* = 54, −42, 28). Please refer to S1 Data for the numerical values underlying this figure. The color scale indicates the *T*-values. Error bars indicate standard errors of the mean.

Finally, we examined the extent to which the clusters showing adaptation to repeated stimulus duration overlapped across experiments 1, 2, and 4 (Fig 9). We found that the clusters in the right SMG overlapped across the three experiments. In existing literature, the right SMG is often labeled as the right temporoparietal junction (TPJ), which refers to the cortex at the intersection of the posterior end of the superior temporal sulcus, the IPL, and the lateral occipital cortex [[23](#_ENREF_23),[24](#_ENREF_24)]. Since a recent TPJ parcellation study [[24](#_ENREF_24)] suggested that the right TPJ is subdivided into three distinct regions depending on the differences in the pattern of anatomical connectivity, we examined which of the subdivisions corresponded to the overlapped area shown in Fig 9 (white area). We first identified the peak coordinates of the clusters for experiments 1, 2, and 4 within the overlapped area (Fig 9), and then the correspondence was evaluated using a connectivity-based parcellation atlas (<http://www.rbmars.dds.nl/CBPatlases.htm>). The result showed that all the peak coordinates within the overlapped area (*x*, *y*, *z* coordinates for experiment 1: 56, −42, 28; experiment 2: 64, −40, 30; experiment 4: 54, −42, 28) were located within the anterior TPJ (TPJa). These findings together strongly indicated that the right IPL (SMG) showed duration-specific adaptation to a broad range of stimulus durations (i.e., 300, 400, 450, and 600 ms), independently of whether the task was to make same-versus-different or shorter-versus-same-versus-longer judgments.

**Fig 9. Overlaps of clusters showing a duration adaptation effect during the time task across experiments.** Colors correspond to the results of each experiment: red for experiment 1, blue for experiment 2, and green for experiment 4. Overlapped areas show mixed colors, as shown in the legend. The clusters in the SMG are magnified at the bottom right.

**Discussion**

Our results demonstrate that neural responses in the right IPL (corresponding to the SMG) exhibit adaptation to repeated presentations of stimuli of the same duration, regardless of whether the participants are engaged in time estimation. This finding provides empirical support for the notion that the right IPL contains neural populations tuned for particular time intervals.

Dysfunction of the right SMG because of stroke [[25](#_ENREF_25)] or virtual lesions created by transcranial magnetic stimulation (TMS) [[26](#_ENREF_26),[27](#_ENREF_27)] results in impairment of time estimation, suggesting that the right SMG is crucial for estimating time intervals. Our finding of duration adaptation in the right SMG is consistent with the findings of these studies and the emerging view of the human SMG as the locus for encoding durations [[26–28](#_ENREF_26)]. Although the representational content of time intervals in the right SMG has been unclear, our findings suggest that time intervals are represented by duration-tuned neural populations in the right SMG.

The involvement of the right IPL in time perception has been reported in a number of neuroimaging studies [[5](#_ENREF_5),[29–39](#_ENREF_29)]. Activation of the right IPL has been shown in response to stimuli of a broad range of durations (sub- and suprasecond) [[35](#_ENREF_35),[37](#_ENREF_37)] and regardless of the nature of the task (perceptual and motor timing) [[40](#_ENREF_40)]. It should be noted, however, that the center of typical activations in these studies was located in the dorsal part of the right IPL (outside of the right SMG) (Talairach coordinates *x*, *y*, *z* = 40, −44, 38, corresponding to 39, −47, 40 in Montreal Neurological Institute (MNI) coordinates; see meta-analysis [[40](#_ENREF_40)]), whereas the right SMG we found was located more inferiorly (MNI coordinates: [58, −42, 30] in experiment 1, [62, −34, 32] in experiment 2, [54, −42, 28] in experiment 4). Moreover, in contrast to the positive BOLD response in the dorsal part of the right IPL shown in the previous studies, overall BOLD response in the right SMG in the present study was negative. Together, these results may suggest that, while the inferior part of the right IPL (i.e., SMG) encodes time by duration-tuned mechanisms, the dorsal part of the right IPL may play a different role in time perception.

The IPL is often labeled as TPJ, which usually refers to the cortex at the intersection of the posterior end of the superior temporal sulcus, the IPL, and the lateral occipital cortex [[23](#_ENREF_23),[24](#_ENREF_24)]. In addition to temporal processing, the right TPJ has been implicated in various types of perception and social cognition, such as reorienting of attention [[41](#_ENREF_41)] and attribution of mental states to others (i.e., a theory of mind [ToM]) [[42](#_ENREF_42),[43](#_ENREF_43)]. However, whether these apparently different types of cognitive functions are commonly subserved by the right TPJ or are processed in the distinct subdivisions of the right TPJ has been a matter of debate. A recent parcellation study proposed that the right TPJ is subdivided into three distinct regions based on diffusion-weighted-imaging tractography: dorsal TPJ, ventral TPJa, and ventral posterior TPJ (TPJp) [[24](#_ENREF_24)]. Moreover, a resting state functional connectivity analysis showed that the TPJa and TPJp are embedded in different functional networks: the right TPJa activity interacted with the bilateral IPL, the ventral prefrontal network, and the anterior insula, which are often associated with ventral attention network [[41](#_ENREF_41),[44](#_ENREF_44)], while the right TPJp interacted with the posterior cingulate, the temporal pole, and the anterior medial prefrontal cortex, which are often implicated in social cognition [[45](#_ENREF_45)]. These lines of evidence suggest that the right TPJ can be subdivided based on anatomical and functional connectivity.

This study, however, also left a possibility that these two different subregions may perform similar neural computation but on different types of information (e.g., social versus perceptual information), and some neural populations located in the adjacent area might not distinguish those different types of information. This possibility seems to be supported by a recent study which demonstrated, using multivoxel pattern analysis, that spatial distance, social distance (i.e., familiarity), and temporal distance are represented by a similar activation pattern in the right TPJ [[46](#_ENREF_46)]. The view of a "shared computational mechanism" is also compatible with the idea that the right TPJ acts as a “reorienting” system for perception and social cognition, such as attribution of another person’s mental states [[23](#_ENREF_23)]. A number of studies have also proposed that the right TPJ is functionally homogeneous by showing that activations in the right TPJ overlap between attention reorienting and ToM tasks [[42](#_ENREF_42),[47](#_ENREF_47)]. Future studies should address whether and how specifically the duration-tuned time representation mechanism is shared or interacts with those reorienting system and social cognition such as ToM.

We found a significant overlap of duration adaptation effect in the right IPL across experiments. The overlapped area corresponded to the right TPJa that was associated with the ventral attention network in the parcellation study [[24](#_ENREF_24)]. A large number of studies have reported that the ventral attention network is involved in stimulus-driven shift of attention. For example, the right TPJ responds to a peripheral target that appears at an unexpected spatial location [[23](#_ENREF_23),[48](#_ENREF_48),[49](#_ENREF_49)] or to an unexpected change in stimulus features [[50–53](#_ENREF_50)]. Based on these previous reports, one might point out that our findings of the greater BOLD responses for the deviant durations than for the repeated duration may reflect violation of prediction, as was shown in a previous face-adaptation study [[54](#_ENREF_54)]. In other words, participants might have expected to see the test stimuli of identical duration as the reference, and the violation of that expectation by the deviant test stimulus might have produced greater activity in the right TPJ than the “same” condition. This alternative account is, however, unlikely to be the sole explanation for our results. In experiments 1 and 2, the number of trials was the same across test durations, and the probability of occurrence for a “different” test duration was much higher than that for the “same” stimuli (80% versus 20%). Under these circumstances, it is more likely that participants expected to see a “different” duration than the “same” duration; thus, the potential effect of prediction error signals for “different” conditions should have been minimized in the present study. This is consistent with a previous study that showed effect of repetition suppression even when the “same” versus “different” response ratio was 1:3 [[55](#_ENREF_55)]. Moreover, in our control experiment with the graded-shape task (experiment 3), we showed that the right IPL was not adapted to the repetition of the same shape, while repetition of the identical durations showed an adaptation effect in this area. The lack of a significant effect of shape adaptation in the right IPL suggests that the graded adaptation for time cannot be explained in terms of the generic concept of prediction errors. Importantly, the task design, task difficulty, number of trials, and number of subjects in experiment 3 were all comparable to the time task in experiments 1 and 2, suggesting that the sensitivity for detecting the adaptation signal was similar across these experiments. Taken together, we suggest that our finding of duration adaptation in the right IPL is more likely to be explained by neural adaptation than by the violation of expectation (i.e., prediction error signal). This conclusion is supported by a previous neuroimaging study that showed insensitivity of the right IPL to the violation of temporal expectation, using a temporal version of the spatial cuing paradigm [[56](#_ENREF_56)].

Training in discrimination of temporal intervals in the range of a few hundreds of milliseconds produces trained-interval-specific improvements in temporal discrimination [[57](#_ENREF_57)]. Intriguingly, the training effect is transferred across sensory modalities [[58](#_ENREF_58)] and from perceptual to motor timing tasks [[59](#_ENREF_59)] in an interval-specific manner. The interval specificity of the learning effect implies that duration-selective neurons mediate the learning of time intervals. A recent fMRI study found stronger activation in the left IPL as well as the left posterior insula following intensive training in duration discrimination in the range of a few hundreds of milliseconds [[60](#_ENREF_60)]. However, changes in right IPL activity were not reported. One possible interpretation for the seemingly discrepant results is that the training effect might be reflected in the tuning, rather than the amplitude, of activity in the right IPL. The adaptation paradigm we reported here offers a method of testing for possible changes in time-related neuronal tuning in future studies.

In our recent voxel-based morphometry (VBM) study [[61](#_ENREF_61)], we reported that individual differences in the ability to discriminate stimulus durations correlated with regional gray-matter (GM) volume: GM volume in the bilateral anterior cerebellum was correlated with the ability to discriminate subsecond (<1 s) durations, while GM volume in the right IPL was correlated with suprasecond (>1 s) durations. The correlation between right IPL GM volumes and suprasecond discrimination thresholds was interpreted as the reflection of the ability to focus attention on continuing to track long time intervals. One might wonder why the fMRI adaptation to the repetition of subsecond durations was found in the right IPL, while correlation between regional GM volumes and discrimination thresholds in the subsecond range was absent in the VBM study [[61](#_ENREF_61)]. To address this point, we scrutinized the data from our previous VBM study [[61](#_ENREF_61)]. We found that, with a slightly more liberal threshold than the stringent one that was used in our VBM study [[61](#_ENREF_61)], the right IPL was correlated with individual differences in subsecond duration discrimination thresholds (MNI coordinates: *x*, *y*, *z* = 57, −27, 44, corresponding to the right SMG; the peak coordinates were identified using the Masked Contrast Images (MASCOI) toolbox with primary and secondary thresholds of *p* < 0.005 and *p* < 0.05, respectively). This was such that a larger GM volume in this area was associated with better duration discrimination performance. This result is consistent with another recent VBM study showing that a larger GM volume in the right TPJ is associated with performance in numerical and continuous quantity (i.e., line length and time estimation in subsecond range) tasks [[62](#_ENREF_62)], although the peak coordinates of the cluster identified in that study were found at a more posterior ventral region than ours. This additional result provides an interesting contrast in the size-performance relationship between subsecond and suprasecond time estimation: better task performance in the subsecond range was associated with larger GM volume, while better performance was associated with smaller GM volume for the suprasecond range. We speculate that this difference might reflect the different functional roles of the right IPL for subsecond and suprasecond time estimation. In our previous VBM study, we attributed the better suprasecond timing performance with smaller IPL to the better ability to focus attention on continuing to track long time intervals. On the other hand, the better performance in the subsecond time estimation associated with greater GM volume in the IPL may reflect finer duration-tuning mechanisms. The duration-specific adaptation in the right IPL suggests that individuals with greater GM volume in this region might have a greater number of neurons tuned to different durations, enhancing the ability to estimate subsecond durations. Testing this potential hypothesis would provide further insight into the anatomo-computational relationship in the right IPL.

We did not find any duration adaptation in the SMA, despite its considerable involvement in various timing tasks, as shown by previous fMRI studies [[40](#_ENREF_40)]. Importantly, a recent electrophysiological study in monkeys reported interval-tuned responses of neurons in the SMA [[63](#_ENREF_63)]. One possible explanation for the lack of duration adaptation in the SMA in our study might be that the interval tuning found in the monkeys was specifically associated with the motor tasks employed by that particular electrophysiological study [[63](#_ENREF_63)]. In contrast, the duration-specific adaptation in our study is likely to reflect more perceptual aspects of time estimation and is independent of the task relevance of duration estimation. Regarding the relationship between the right IPL and SMA, a recent TMS study proposed a feed-forward mechanism of temporal information from the right SMG to the SMA [[27](#_ENREF_27)]. That study showed that TMS over the right SMG influenced the subjective perception of time intervals, as they reported earlier [[26](#_ENREF_26)], and that the degree of time dilation was reflected in the contingent negative variation (CNV) amplitude recorded from the frontocentral site in the measurement window around the stimulus offsets [[27](#_ENREF_27)]. On the basis of an earlier report showing a positive correlation between the CNV amplitude and SMA activity as measured by BOLD signals [[64](#_ENREF_64)], Wiener and colleagues (2012) suggested that there was a feed-forward mechanism of temporal information from the right SMG to the SMA. Intriguingly, it has also been shown recently by electroencephalography (EEG) that the amplitude of the potentials evoked by the offset of comparison intervals reflects subjective time intervals better than does the amplitude of the CNV [[65](#_ENREF_65)]. Our findings, together with these previous reports, suggest that duration information is primarily encoded in the duration-tuned neural populations in the right IPL, regardless of the task; the information is then transferred to the SMA for task-specific processing.

Although a considerable number of previous studies reported the involvement of the basal ganglia and cerebellum in time perception [[40](#_ENREF_40),[66](#_ENREF_66),[67](#_ENREF_67)], neither of these regions were found to show consistent adaptation across different durations. The basal ganglia showed adaptation only to the repetition of 400 ms (experiment 1, see S1 Table), but this was not replicated for 600 ms in experiment 2. These results may suggest that the duration tuning of basal ganglia is limited to the durations of around 400 ms; however, this notion requires further investigation. Although a previous theoretical study has proposed that the cerebellum may contain neurons tuned for duration [[11](#_ENREF_11)], a duration adaptation effect was not found in the cerebellum in our study. However, it is important to note that the cerebellum was not consistently covered by the field of view in our fMRI experiments except for experiment 4, and therefore, the lack of positive findings in the cerebellum should be taken with caution. Although the cerebellum was covered in experiment 4, further investigations with a broader range of reference durations would be needed to draw a firm conclusion on this issue.

Our results showed negative BOLD responses at the offset of the test stimuli in the right IPL. The negative BOLD response indicates a reduction in overall neural activity [[68](#_ENREF_68)]. While a deactivation rather than a reduction in the degree of BOLD activation may seem puzzling, similar deactivation patterns for orientation-tuned fMRI adaptation in the visual cortex have previously been reported [[69](#_ENREF_69)]. In that study, Fang et al. investigated the effect of neural adaptation effect for orientation tuning in the visual cortex following brief adaptation versus prolonged adaptation. The study found that the neural adaptation in the visual cortex was related to suppression of the BOLD signal for repetitions with the same orientation and to a graded increase of the BOLD response as the difference in adaptor and test stimulus orientation increased. Similarly to the present study, they showed a negative BOLD response when the same orientation was repeated (0°) or when the orientation of the test stimulus was only slightly different (7.5°) from that of the adaptor. Fang et al. interpreted this negative response as “[…] maybe attributed to the overlapping neural populations tuned to 0 and 7.5°” [[69](#_ENREF_69)]. The idea of the overlapped neural populations is also consistent with the computational model of duration channels [12].

We speculate that the overall negative responses observed in the present study may reflect suppression of neural activity due to attentional focus on the duration judgment task. Previous studies have reported that the right TPJ shows strong deactivations during attentionally demanding visual tasks [[23](#_ENREF_23),[70–7](#_ENREF_71)2]. Since the right TPJ has been considered to act as a “circuit breaker” that interrupts ongoing processes when a potentially task-relevant stimulus appears [[41](#_ENREF_41)], the deactivation of the right TPJ has been proposed to play a role in preventing attention from reorienting to task-irrelevant information when performing an attentionally demanding task [[23](#_ENREF_23), 71,72]. Time perception is generally susceptible to interference from task-irrelevant stimulus properties (e.g., [12,[34](#_ENREF_34), [73–](#_ENREF_74)77]) and thus likely to require attention. The relatively low task performance in the present study also suggests that attentional demand was high during the time task in the present study. The observed negative BOLD response in the right IPL could therefore be attributed to inhibition of task-irrelevant inputs for accurate time estimation. One possible interpretation for the negative BOLD response in our study is that this deactivation stems from a mixture of neuronal populations that are deactivated during the task and neural populations that are activated for specific durations. In this interpretation, the duration-tuned neural populations are adapted by the repetition of the same duration, whereas other neurons, unrelated to time estimation, are suppressed during the task, causing the overall negative BOLD responses in the right IPL. This interpretation is in line with previous studies that attributed negative BOLD responses in the right TPJ during other types of visual tasks to the mixture of increased and decreased activities of heterogeneous neuronal populations in the right TPJ [[23](#_ENREF_23),71]. Importantly, the negative BOLD response at the offset of the test stimuli was also found in the graded-shape discrimination task in experiment 3 in which the task design and difficulty was comparable to the time task in experiments 1 and 2. Nevertheless, variation in the degree of BOLD reduction (i.e., fMRI adaptation) was uniquely found for the repetition of identical duration but not for the repetition of a shape. These results further support our interpretation that the negative BOLD response at the offset of the test duration found in the right IPL is related to the attention focus in general, while graded adaptation effect was unique to the repetition of the same duration.

It has been shown that extensive adaptation to repetitions of identical durations (e.g., ~100 repetitions) can produce perceptual changes in perceived duration (“aftereffect”) [12]. In contrast, the paired presentation paradigm with a single presentation of an adapter does not produce a perceptual change in duration as shown by a previous psychophysical study (see Discussion in [12]). Although our fMRI adaptation paradigm and previous psychophysical studies of time adaptation [12,[13](#_ENREF_13)] indicate the existence of duration-tuned neural representations, further investigation is required to determine whether the right IPL identified in the present study mediates the psychophysically observed aftereffect. Intriguingly, previous studies of orientation adaptation demonstrated that a prolonged presentation of an adapter produced an fMRI adaptation across multiple visual areas, whereas adaptation to a briefly presented stimulus produced adaptation only in the higher visual cortex [[69](#_ENREF_69),78]. These neuroimaging studies in orientation adaptation suggest that differences in the strength of adaptation could produce different fMRI adaptation. Direct comparison between the two different adaptation paradigms (i.e., single presentation versus 100 times repetition of the same duration) would provide further insight into the relationship between duration-tuned representations in the right IPL and the psychophysical duration aftereffect.

Here, we carefully ruled out potential confounding factors and replicated the main findings by using multiple experimental paradigms and four standard durations. Our findings of duration adaptation in the right IPL are attributable neither to general decision-making processes (experiments 1 and 2) nor to graded manipulation of a stimulus feature (experiment 3). Moreover, we confirmed that adaptation was induced by the duration of the reference stimulus and not by the fixed interval between the reference and test stimuli (experiment 4). Differences in task difficulty between conditions or experiments were equated and thus should not have accounted for our findings of duration adaptation (experiments 3 and 4). The replication of duration adaptation in the right IPL across the experiments strongly corroborates our conclusion that this area represents the durations of salient events in a duration-selective manner. On the other hand, repetition suppression in the other brain areas such as the PTC and prefrontal cortex were not reproduced across experiments in the present study. One possible interpretation for this experiment specific effect is that such apparent repetition suppression was the result of the potential confounding factors listed above (e.g., general decision-making processes, graded manipulation of a stimulus feature, and fixed interval between the reference and test stimuli). Alternatively, neurons in those areas might specifically be tuned for a very narrow range of duration used in that particular experiment. Future studies applying single-unit recording technique for those areas (e.g., PTC and prefrontal cortex) in nonhuman primates could provide clear insight into this issue.

Before our work, neural correlates of time perception had been found in the form of gradual changes in neural activity over time, such as in the hazard rate of elapsed time [[3](#_ENREF_3),[5](#_ENREF_5)] or in the form of ramping activity over time [[6](#_ENREF_6),79,80]. Although several theoretical models have assumed that duration is represented by populations of neurons that fire selectively for the durations to which they are tuned [[8–10](#_ENREF_8)], empirical evidence for such neurons in humans has until now been missing. Only a handful of electrophysiological studies in animals have reported the existence of neurons that show duration-selective responses to time intervals of a few hundreds of milliseconds; these neurons have been located in the striatum and prefrontal cortex [81] and the SMA [[63](#_ENREF_63)] in monkeys and in the visual cortex in cats [82]. Our findings suggest that such duration-selective neurons exist in the human IPL.

**Materials and Methods**

**Ethics Statement**

All participants gave written informed consent. Experiments 1, 2, and 3 were approved by the University College London (London, United Kingdom) ethics committee, and experiment 4 was approved by the National Institute for Physiological Sciences (Okazaki, Japan) ethics committee.

**Participants**

In total, 55 healthy, right-handed volunteers participated in the fMRI experiments. Seventeen volunteers participated in experiment 1. In experiment 2, another group of 17 volunteers was recruited. These 17 volunteers in experiment 2 also participated in experiment 3. In experiment 4, a new group of 21 volunteers participated. Data of five participants in experiment 1, three participants in experiment 2, three participants in experiment 3, and one participant in experiment 4 were excluded from data analyses because of poor task performance in the duration or shape discrimination task (binomial test, *p* > 0.05). Moreover, one more participant was excluded from the data analysis of experiment 3 because of partial signal loss caused by a large movement of the head. Therefore, data from the remaining 12 participants (five male and seven female, age range 19 to 28 y) in experiment 1, 14 participants in experiment 2 (five male and nine female, age range 21 to 30 y), 13 participants in experiment 3 (five male and eight female, age range 21 to 29 y), and 20 participants (nine male and 11 female, age range 18 to 29 y) in experiment 4 were analyzed.

**Task and Stimuli**

**Experiments 1 and 2.** The task was either (1) to discriminate whether the durations of two sequentially presented visual stimuli were the same or different (time task) or (2) to discriminate whether the shapes of the two stimuli were the same or different (shape task) (Fig 1A).

Each participant completed a total of four runs of fMRI scans (9 min 25 s and 9 min 48 s per run for experiment 1 and experiment 2, respectively), each of which comprised 16 blocks (eight blocks for each task); each block consisted of five trials. An instruction cue (duration 1.5 s) indicated whether the task in the following five trials was a time (“Time”) or a shape (“Shape”) task. In both tasks, a trial started with the presentation of a fixation cross for 1.5 s, followed by a reference and a test stimulus that were presented sequentially at the center of the screen with an interval of 0.5 s. Then, a fixation cross was presented for a variable duration of time (1 to 3 s, with a 0.5-s step), followed by a response cue. The response cue “S D” indicated that the subject’s right index finger was assigned to “same” and the right middle finger to “different” responses; in contrast, “D S” indicated that the right index finger was assigned to “different” and the middle finger to “same” responses. These cues were randomly presented across trials. Participants were requested to respond within the response period of 1 s. Interblock intervals were 2 s, and block durations (i.e., intervals between onset of instruction cue presented at the beginning of each block and the offset of the response cue at the last trial in that block) were varied within the range of 26.5–34.7 s (experiment 1) and 28.5–37.5 s (experiment 2). Before the fMRI session, participants were instructed to fixate on the center of the screen throughout the run and to ignore the task-irrelevant dimension (i.e., the shape of the stimulus during the time task and the stimulus duration during the shape task). The blocks for the time task and for the shape task were mixed in each run, and the presentation order of these types of block was randomized.

The reference and test stimuli were a square or a circle presented on a black background. The total areas of the stimuli were equated so as to control their physical properties. The stimulus durations for the reference and test stimuli differed between experiment 1 and experiment 2. The duration of the reference stimulus presentation was fixed at 400 ms for experiment 1 and 600 ms for experiment 2, whereas the duration of the test stimulus was varied on five levels (167, 283, 400, 533, and 650 ms for experiment 1 and 250, 433, 600, 783, and 967 ms for experiment 2). There were four combinations of shape pairs for the reference and test stimuli, namely square-square (SS), circle-circle (CC), square-circle (SC), and circle-square (CS). Therefore, each experiment consisted of 20 different types of trial (5 levels of test stimulus duration × 4 combinations of shape), and each of them was presented four times in each run (Half were presented in the time task, and the other half in the shape task.).

**Experiment 3.** The task was to discriminate whether the widths of two sequentially presented visual stimuli (an oval-shaped reference and a width-varying oval-shaped test stimulus) were the same or different (graded-shape task) (Fig 4A).

The experiment consisted of two runs (11 min 28 s per run), each of which had 20 blocks of five trials. The durations of the reference and test stimuli, as well as the durations of the fixation cross, the instruction cue (only “Shape” was presented), and the response cue (“S D” or “D S”) and interblock intervals were the same as in experiment 1. Block durations were varied within the range of 26.7–34.8 s. The visual stimulus was a white oval presented on a black background. The reference stimulus was identical across all trials (width of oval = 60 pixels), whereas the test stimuli varied in width on five levels, namely 50 pixels (very narrow [VN]), 56 pixels (narrow [N]), 60 pixels (same [S]), 64 pixels (wide [W]), and 70 pixels (very wide [VW]). In total, the experiment had 25 types of trial (5 levels of test stimulus duration × 5 levels of test stimulus width), and each of them was presented four times in each run.

**Experiment 4.** The task was to discriminate whether the duration of the second (test) stimulus of two sequentially presented visual stimuli was shorter, longer, or the same compared with the duration of the first (reference) stimulus (time task) (Fig 7A).

Each participant completed a total of five runs of fMRI scans (7 min 52 s per run), each of which comprised 12 blocks of four trials: six blocks with a reference (adaptor) of 300 ms and six blocks with a reference (adaptor) of 450 ms. Each block began with an instruction cue of 1.5 s (Only “Time” was presented.). A trial started with the presentation of a fixation cross for 1.5 s, followed by a reference and a test stimulus that were presented sequentially at the center of the screen with a variable ISI (1 to 2.5 s, with a 0.5-s step). Then, a fixation cross was presented for a variable duration (2 to 3.5 s, with a 0.5-s step), followed by a response cue. The response cue consisted of “+,” “-,” and “=,” corresponding to “longer,” “shorter,” and the “same” responses, respectively. The spatial locations of these cues indicated the assignment of each response to one of the three buttons on the button box. For example, “+ - =” indicated that the right index finger was now assigned to the response “longer,” the middle finger to “shorter,” and the ring finger to “same.” The cues (i.e., “+ - =,” “+ = -,” “- + =,” “- = +,” “= + -,” or “= - +”) were randomly presented across the trials. Participants were requested to respond within the response period of 2 s. Before the fMRI session, participants were instructed to fixate on the center of the screen throughout the session. The blocks with 300-ms reference and with 450-ms reference were mixed in each run, and the presentation order of these types of block was randomized. Interblock intervals were 1 s, and block durations were varied within the range of 33.5–40.3 s.

The reference and test stimuli were white squares presented on a black background. The sets of test stimulus durations in the 300-ms and 450-ms reference blocks differed. The test stimulus durations when the 300-ms reference blocks were used were 200 ms, 300 ms, and 450 ms, whereas those when the 450-ms reference blocks were used were 300 ms, 450 ms, and 667 ms. Therefore, this experiment consisted of six different types of trial (2 reference stimulus durations × 3 levels of test stimulus duration), each of which was presented eight times in each run.

**Behavioral Data Analysis**

The proportions of the “same” responses in the time task were fitted by a Gaussian function for individuals’ behavioral data. A one-sample *t*-test (α = 0.05) was performed to examine whether the center of the Gaussian was shifted from the reference durations (experiments 1, 2, and 4) or the reference stimulus width (experiment 3). A one-way ANOVA was performed to compare the task difficulties (i.e., accuracy) across experiments (α = 0.05). Also, a one-way repeated measures ANOVA was performed to compare the accuracy across conditions in experiment 4 (α = 0.05). Degree of freedom was corrected using Greenhouse-Geisser estimates of sphericity when Mauchly’s test indicated that the assumption of sphericity was violated.

**Functional MRI Data Acquisition**

Visual stimuli were projected onto a half-transparent screen by an LCD projector running at 60 Hz. The screen was viewed through a mirror mounted on the head coil. Psychtoolbox (http://psychtoolbox.org) implemented on MATLAB software (Mathworks, Natick, Massachusetts) was used to present the stimuli in experiments 1, 2, and 3, and Presentation Software (Neurobehavioral System, Berkeley, California) was used in experiment 4.

MR images for experiments 1, 2, and 3 were acquired with a 1.5-T MRI scanner (Avanto, Siemens, Munich, Bavaria, Germany). Data were acquired by using a 32-channel head coil. Time-course series of 175 (experiment 1), 182 (experiment 2), and 213 (experiment 3) volumes were acquired by using ascending T2*-weighted gradient-echo echo-planar imaging (EPI) sequences. To cover the entire cerebral cortex and basal ganglia, each volume consisted of 38 oblique slices, with 3.0 × 3.0 mm resolution, 2.0 mm thickness, and a 1.0-mm slice gap. The cerebellar cortices were not covered. The time interval between two successive acquisitions of the same slice was 3,230 ms, with a flip angle of 90° and a 50-ms echo time. The field of view was 192 × 192 mm. The digital in-plane resolution was 64 × 64 pixels, with a pixel dimension of 3.0 × 3.0 mm. High-resolution whole-brain MR images were also obtained by using a T1-weighted three-dimensional (3-D) magnetization-prepared rapid acquisition gradient-echo (MPRAGE) sequence (voxel size = 1.0 × 1.0 × 1.0 mm). The first six volumes of each fMRI run were discarded because of unsteady magnetization, and the remaining 169, 176, and 207 volumes per run (a total of 676, 704, and 414 volumes per participant) were used for analysis in experiment 1, experiment 2, and experiment 3, respectively.

MR images for experiment 4 were acquired with a 3-T MRI scanner (Allegra, Siemens, Munich, Bavaria, Germany). A time-course series of 236 volumes was acquired by using ascending T2*-weighted gradient-echo EPI sequences. To cover the entire cerebral and cerebellar cortex and basal ganglia, each volume consisted of 34 oblique slices, with 3.0 × 3.0 mm resolution, 3.5 mm thickness, and a 0.56-mm slice gap. The time interval between two successive acquisitions of the same slice was 2,000 ms, with a flip angle of 80° and a 30-ms echo time. The field of view was 192 × 192 mm. The digital in-plane resolution was 64 × 64 pixels, with a pixel dimension of 3.0 × 3.0 mm. High-resolution whole-brain MR images were also obtained by using a T1-weighted 3-D MPRAGE sequence (voxel size = 1.0 × 1.0 × 1.0 mm). The first five volumes of each fMRI run were discarded because of unsteady magnetization, and the remaining 231 volumes per session (a total of 1,155 volumes per participant) were used for the analysis.

**Preprocessing of fMRI Data**

The fMRI data were analyzed by using statistical parametric mapping software (SPM8; http://www.fil.ion.ucl.ac.uk/spm/) implemented in MATLAB. The MR images were preprocessed at the individual level. Following realignment of the fMRI data, the high-resolution 3-D T1-weighted MR images were coregistered to the fMRI data. The coregistered T1-weighted images were normalized against the MNI T1 template, and the same parameters were applied to all of the fMRI data. The anatomically normalized fMRI data were then smoothed in three dimensions by using an 8-mm full-width-at-half-maximum Gaussian kernel. Before the coregistration, slice-timing correction was applied only to the data of experiment 4, because the TR of fMRI scan was short enough (< 3 s) to apply the correction.

**Statistical Analysis of fMRI Data**

**Experiments 1 and 2.** We constructed two general linear models (GLMs) and applied them to the same datasets in experiment 1 and experiment 2. The first model was aimed at evaluating the effects of adaptation to time while performing the time task and adaptation to shape while performing the shape task. The second model was designed to evaluate whether the effect of adaptation to time would appear while performing the shape task. For these purposes, in the first model, the regressors were set at the onsets of the instruction, the reference stimulus presentation for the time task, the reference stimulus presentation for the shape task, and the response phase, as well as at the time of offset of the test stimulus presentations. There were nine independent regressors for the offsets of the test stimulus presentations, namely five levels of duration conditions for the time task trials, along with the SS, CC, SC, and CS conditions for the shape task trials. In total, therefore, 13 regressors were set for each run.

In the second model, there were ten independent regressors for the offsets of the test stimulus presentations, namely five levels of duration conditions for the time task trial and five for the shape task trial. The regressors for the onsets of the instruction, the reference stimulus presentation for the time task, the reference stimulus presentation for the shape task, and the response phase were also set as in the first model. In total, therefore, 14 regressors were set for each run of fMRI scanning. The duration of all regressors in both models in SPM8 was set to zero. The signal time course of each participant was modeled with hemodynamic-response functions, high-pass filtering (256 s), and session effects.

The summary data for each participant were incorporated into a group-level analysis using a random effects model, separately for the first and the second model. The parameter estimates in the individual analysis constituted “contrast” images, which were used for the group analysis. To detect brain regions that showed fMRI adaptation effects, the contrast images for each condition were obtained via individual analysis representing the event-related change in MR signals at the offset of the test stimuli. For these contrast images, a full factorial analysis was performed for every voxel within the brain, in order to obtain population inferences for the effect of duration adaptation and the effect of shape adaptation (“same” versus “different” conditions). To detect the duration adaptation effect, we applied a parametric contrast according to the perceptual duration change between the reference and the test stimuli. In the present study, the deviation ratio was used as a proxy of perceptual discriminability of durations. The deviation ratio [(longer duration − shorter duration) / shorter duration] for each set of stimulus durations was 1.4 (167 ms), 0.41 (283 ms), 0 (400 ms), 0.33 (533 ms), and 0.63 (650 ms) for experiment 1 and 1.4 (250 ms), 0.39 (433 ms), 0 (600 ms), 0.31 (783 ms), and 0.61 (967 ms) for experiment 2 (Fig 1C). The means of these values were adjusted to zero and then the mean-adjusted values were applied as the contrast values. Unless otherwise noted, a statistical threshold of *p* < 0.05 FWE-corrected at the cluster level (defined as *p* < 0.001 uncorrected at the voxel level) was used as the criterion for statistical significance.

**Experiment 3.** We constructed a GLM to evaluate the effect of adaptation to stimulus width while subjects were performing the graded-shape task. Similarly to experiments 1 and 2, we also constructed another GLM to evaluate whether the effect of adaptation to time would appear while performing the graded-shape task. The regressors were set at the onsets of the instruction, the reference stimulus presentation, and the response phases, as well as at the offsets of the test stimulus presentations. In the first model, there were five independent regressors for the offsets of the test stimulus presentations; these corresponded to the five graded oval widths. In the second model, these instead corresponded to the five levels of duration conditions. In total, therefore, eight regressors were set for each run in both models. The duration of all regressors in SPM8 was set to zero. The signal time course of each participant was modeled with hemodynamic-response functions, high-pass filtering (256 s), and session effects.

In the group-level analysis with a random effects model, we performed full factorial analyses in order to obtain population inferences for the effect of graded-shape adaptation and the effect of duration adaptation. To detect the graded-shape adaptation effect, we applied a parametric contrast according to the perceptual width change between the reference and the test stimuli. Similarly to the time tasks in experiments 1 and 2, we used deviation ratio as a proxy of perceptual discriminability of widths. The deviation ratio for each test condition was 0.2 (VN), 0.07 (N), 0 (S), 0.07 (W), and 0.17 (VW) (Fig 4C). To detect the duration adaptation effect, we applied the same parametric contrast as experiment 1 because the deviation ratios for the test stimulus durations in this experiment were the same as in experiment 1. The means of these values were adjusted to zero and then the mean-adjusted values were applied as the contrast values. Unless otherwise noted, the same statistical threshold as in experiments 1 and 2 was used as the criterion for statistical significance.

**Experiment 4.** We constructed a GLM to evaluate the effects of adaptation to time while subjects were performing the time task with two different adaptor durations. The regressors were set at the onsets of the instruction, the 300-ms reference stimulus presentation, the 450-ms reference stimulus presentation, and the response phases, as well as at the offsets of the test stimulus presentations. There were six independent regressors for the offsets of the test stimulus presentations, namely three test durations that followed the 300-ms reference stimulus and three test durations that followed the 450-ms reference stimulus. In total, therefore, ten regressors were set for each run. The duration of all regressors in SPM8 was set to zero. The signal time course of each participant was modeled with hemodynamic-response functions, high-pass filtering (256 s), and session effects.

In the group-level analysis with a random effects model, we performed a full factorial analysis in order to obtain population inferences for the effect of duration adaptation. To detect the duration adaptation effect, we applied a parametric contrast according to the deviation ratio between the reference and test stimuli. The deviation ratio for each test duration that followed the reference stimulus of 300 ms was 0.5 (200 ms), 0 (300 ms), and 0.5 (450 ms), and the ratios for the durations that followed the reference stimulus of 450 ms were 0.5 (300 ms), 0 (450 ms), and 0.5 (667 ms) (Fig 7C). The means of these values were adjusted to zero and then the mean-adjusted values were applied as the contrast values. Unless otherwise noted, the same statistical threshold as used in experiments 1, 2, and 3 was used as the criterion for statistical significance.**Acknowledgments**

The authors thank Dr. T. Kochiyama for supporting statistical analysis.

**References**

1. Buhusi CV, Meck WH. What makes us tick? Functional and neural mechanisms of interval timing. Nat Rev Neurosci. 2005;6(10):755-65.

2. Mauk MD, Buonomano DV. The neural basis of temporal processing. Annu Rev Neurosci. 2004;27:307-40.

3. Janssen P, Shadlen MN. A representation of the hazard rate of elapsed time in macaque area LIP. Nat Neurosci. 2005;8(2):234-41.

4. Bueti D, Bahrami B, Walsh V, Rees G. Encoding of temporal probabilities in the human brain. J Neurosci. 2010;30(12):4343-52.

5. Bueti D, Macaluso E. Auditory temporal expectations modulate activity in visual cortex. Neuroimage. 2010;51(3):1168-83.

6. Leon MI, Shadlen MN. Representation of time by neurons in the posterior parietal cortex of the macaque. Neuron. 2003;38(2):317-27.

7. Wittmann M, Simmons AN, Aron JL, Paulus MP. Accumulation of neural activity in the posterior insula encodes the passage of time. Neuropsychologia. 2010;48(10):3110-20.

8. Matell MS, Meck WH. Cortico-striatal circuits and interval timing: coincidence detection of oscillatory processes. Brain Res Cogn Brain Res. 2004;21(2):139-70.

9. Desmond JE, Moore JW. Adaptive timing in neural networks: the conditioned response. Biol Cybern. 1988;58(6):405-15.

10. Buonomano DV, Laje R. Population clocks: motor timing with neural dynamics. Trends Cogn Sci. 2010;14(12):520-7.

11. Ivry RB. The representation of temporal information in perception and motor control. Curr Opin Neurobiol. 1996;6(6):851-7.

12. Heron J, Aaen-Stockdale C, Hotchkiss J, Roach NW, McGraw PV, Whitaker D. Duration channels mediate human time perception. Proc Biol Sci. 2012;279(1729):690-8.

13. Becker MW, Rasmussen IP. The rhythm aftereffect: support for time sensitive neurons with broad overlapping tuning curves. Brain Cogn. 2007;64(3):274-81.

14. Grill-Spector K, Henson R, Martin A. Repetition and the brain: neural models of stimulus-specific effects. Trends Cogn Sci. 2006;10(1):14-23.

15. Krekelberg B, Boynton GM, van Wezel RJ. Adaptation: from single cells to BOLD signals. Trends Neurosci. 2006;29(5):250-6.

16. Malach R. Targeting the functional properties of cortical neurons using fMR-adaptation. Neuroimage. 2012;62(2):1163-9.

17. Piazza M, Izard V, Pinel P, Le Bihan D, Dehaene S. Tuning curves for approximate numerosity in the human intraparietal sulcus. Neuron. 2004;44(3):547-55.

18. Melgire M, Ragot R, Samson S, Penney TB, Meck WH, Pouthas V. Auditory/visual duration bisection in patients with left or right medial-temporal lobe resection. Brain Cogn. 2005;58(1):119-24.

19. Dale CL, Gratton G, Gibbon J. Event-related brain potentials isolate the motor component in a tapping task. Neuroreport. 2001;12(14):3015-8.

20. Wearden JH, Bray S. Scalar timing without reference memory? Episodic temporal generalization and bisection in humans. The Quarterly journal of experimental psychology B, Comparative and physiological psychology. 2001;54(4):289-309.

21. Gibbon J, Church RM, Meck WH. Scalar timing in memory. Ann N Y Acad Sci. 1984;423:52-77.

22. Brannon EM, Libertus ME, Meck WH, Woldorff MG. Electrophysiological measures of time processing in infant and adult brains: Weber's Law holds. J Cogn Neurosci. 2008;20(2):193-203.

23. Corbetta M, Patel G, Shulman GL. The reorienting system of the human brain: from environment to theory of mind. Neuron. 2008;58(3):306-24.

24. Mars RB, Sallet J, Schuffelgen U, Jbabdi S, Toni I, Rushworth MF. Connectivity-based subdivisions of the human right "temporoparietal junction area": evidence for different areas participating in different cortical networks. Cereb Cortex. 2012;22(8):1894-903.

25. Harrington DL, Haaland KY, Hermanowicz N. Temporal processing in the basal ganglia. Neuropsychology. 1998;12(1):3-12.

26. Wiener M, Hamilton R, Turkeltaub P, Matell MS, Coslett HB. Fast forward: supramarginal gyrus stimulation alters time measurement. J Cogn Neurosci. 2010;22(1):23-31.

27. Wiener M, Kliot D, Turkeltaub PE, Hamilton RH, Wolk DA, Coslett HB. Parietal influence on temporal encoding indexed by simultaneous transcranial magnetic stimulation and electroencephalography. J Neurosci. 2012;32(35):12258-67.

28. Merchant H, Harrington DL, Meck WH. Neural basis of the perception and estimation of time. Annu Rev Neurosci. 2013;36:313-36.

29. Bueti D, Macaluso E. Physiological correlates of subjective time: evidence for the temporal accumulator hypothesis. Neuroimage. 2011;57(3):1251-63.

30. Bueti D, Bahrami B, Walsh V. Sensory and association cortex in time perception. J Cogn Neurosci. 2008;20(6):1054-62.

31. Coull JT, Hwang HJ, Leyton M, Dagher A. Dopamine precursor depletion impairs timing in healthy volunteers by attenuating activity in putamen and supplementary motor area. J Neurosci. 2012;32(47):16704-15.

32. Coull JT, Vidal F, Nazarian B, Macar F. Functional anatomy of the attentional modulation of time estimation. Science. 2004;303(5663):1506-8.

33. Harrington DL, Boyd LA, Mayer AR, Sheltraw DM, Lee RR, Huang M, et al. Neural representation of interval encoding and decision making. Brain Res Cogn Brain Res. 2004;21(2):193-205.

34. Hayashi MJ, Kanai R, Tanabe HC, Yoshida Y, Carlson S, Walsh V, et al. Interaction of numerosity and time in prefrontal and parietal cortex. J Neurosci. 2013;33(3):883-93.

35. Lewis PA, Miall RC. Brain activation patterns during measurement of sub- and supra-second intervals. Neuropsychologia. 2003;41(12):1583-92.

36. Maquet P, Lejeune H, Pouthas V, Bonnet M, Casini L, Macar F, et al. Brain activation induced by estimation of duration: a PET study. Neuroimage. 1996;3(2):119-26.

37. Morillon B, Kell CA, Giraud AL. Three stages and four neural systems in time estimation. J Neurosci. 2009;29(47):14803-11.

38. Schubotz RI, Friederici AD, von Cramon DY. Time perception and motor timing: a common cortical and subcortical basis revealed by fMRI. Neuroimage. 2000;11(1):1-12.

39. Schubotz R, von Cramon D. Interval and ordinal properties of sequences are associated with distinct premotor areas. Cereb Cortex. 2001;11(3):210-22.

40. Wiener M, Turkeltaub P, Coslett HB. The image of time: a voxel-wise meta-analysis. Neuroimage. 2010;49(2):1728-40.

41. Corbetta M, Shulman GL. Control of goal-directed and stimulus-driven attention in the brain. Nat Rev Neurosci. 2002;3(3):201-15.

42. Decety J, Lamm C. The role of the right temporoparietal junction in social interaction: how low-level computational processes contribute to meta-cognition. Neuroscientist. 2007;13(6):580-93.

43. Saxe R. Uniquely human social cognition. Curr Opin Neurobiol. 2006;16(2):235-9.

44. Himmelbach M, Erb M, Karnath HO. Exploring the visual world: the neural substrate of spatial orienting. Neuroimage. 2006;32(4):1747-59.

45. Saxe R, Kanwisher N. People thinking about thinking peopleThe role of the temporo-parietal junction in “theory of mind”. NeuroImage. 2003;19(4):1835-42.

46. Parkinson C, Liu S, Wheatley T. A Common Cortical Metric for Spatial, Temporal, and Social Distance. Journal of Neuroscience. 2014;34(5):1979-87.

47. Mitchell JP. Activity in right temporo-parietal junction is not selective for theory-of-mind. Cereb Cortex. 2008;18(2):262-71.

48. Corbetta M, Kincade JM, Ollinger JM, McAvoy MP, Shulman GL. Voluntary orienting is dissociated from target detection in human posterior parietal cortex. Nat Neurosci. 2000;3(3):292-7.

49. Kincade JM, Abrams RA, Astafiev SV, Shulman GL, Corbetta M. An event-related functional magnetic resonance imaging study of voluntary and stimulus-driven orienting of attention. J Neurosci. 2005;25(18):4593-604.

50. Downar J, Crawley AP, Mikulis DJ, Davis KD. A multimodal cortical network for the detection of changes in the sensory environment. Nat Neurosci. 2000;3(3):277-83.

51. Marois R, Leung HC, Gore JC. A stimulus-driven approach to object identity and location processing in the human brain. Neuron. 2000;25(3):717-28.

52. Bledowski C, Prvulovic D, Goebel R, Zanella FE, Linden DE. Attentional systems in target and distractor processing: a combined ERP and fMRI study. Neuroimage. 2004;22(2):530-40.

53. Stevens MC, Calhoun VD, Kiehl KA. Hemispheric differences in hemodynamics elicited by auditory oddball stimuli. Neuroimage. 2005;26(3):782-92.

54. Summerfield C, Trittschuh EH, Monti JM, Mesulam MM, Egner T. Neural repetition suppression reflects fulfilled perceptual expectations. Nat Neurosci. 2008;11(9):1004-6.

55. Larsson J, Smith AT. fMRI repetition suppression: neuronal adaptation or stimulus expectation? Cereb Cortex. 2012;22(3):567-76.

56. Coull JT, Frith CD, Buchel C, Nobre AC. Orienting attention in time: behavioural and neuroanatomical distinction between exogenous and endogenous shifts. Neuropsychologia. 2000;38(6):808-19.

57. Wright BA, Buonomano DV, Mahncke HW, Merzenich MM. Learning and generalization of auditory temporal-interval discrimination in humans. J Neurosci. 1997;17(10):3956-63.

58. Nagarajan SS, Blake DT, Wright BA, Byl N, Merzenich MM. Practice-related improvements in somatosensory interval discrimination are temporally specific but generalize across skin location, hemisphere, and modality. J Neurosci. 1998;18(4):1559-70.

59. Meegan DV, Aslin RN, Jacobs RA. Motor timing learned without motor training. Nat Neurosci. 2000;3(9):860-2.

60. Bueti D, Lasaponara S, Cercignani M, Macaluso E. Learning about time: plastic changes and interindividual brain differences. Neuron. 2012;75(4):725-37.

61. Hayashi MJ, Kantele M, Walsh V, Carlson S, Kanai R. Dissociable neuroanatomical correlates of subsecond and suprasecond time perception. J Cogn Neurosci. 2014;26(8):1685-93.

62. Cappelletti M, Chamberlain R, Freeman ED, Kanai R, Butterworth B, Price CJ, et al. Commonalities for Numerical and Continuous Quantity Skills at Temporo-parietal Junction. J Cogn Neurosci. 2014;26(5):986-99.

63. Merchant H, Perez O, Zarco W, Gamez J. Interval Tuning in the Primate Medial Premotor Cortex as a General Timing Mechanism. Journal of Neuroscience. 2013;33(21):9082-96.

64. Nagai Y, Critchley HD, Featherstone E, Fenwick PB, Trimble MR, Dolan RJ. Brain activity relating to the contingent negative variation: an fMRI investigation. Neuroimage. 2004;21(4):1232-41.

65. Kononowicz TW, van Rijn H. Decoupling Interval Timing and Climbing Neural Activity: A Dissociation between CNV and N1P2 Amplitudes. J Neurosci. 2014;34(8):2931-9.

66. Koch G, Oliveri M, Caltagirone C. Neural networks engaged in milliseconds and seconds time processing: evidence from transcranial magnetic stimulation and patients with cortical or subcortical dysfunction. Philos Trans R Soc Lond B Biol Sci. 2009;364(1525):1907-18.

67. Coull JT, Cheng RK, Meck WH. Neuroanatomical and neurochemical substrates of timing. Neuropsychopharmacology : official publication of the American College of Neuropsychopharmacology. 2011;36(1):3-25.

68. Shmuel A, Augath M, Oeltermann A, Logothetis NK. Negative functional MRI response correlates with decreases in neuronal activity in monkey visual area V1. Nat Neurosci. 2006;9(4):569-77.

69. Fang F, Murray SO, Kersten D, He S. Orientation-tuned FMRI adaptation in human visual cortex. J Neurophysiol. 2005;94(6):4188-95.

70. Marois R, Yi DJ, Chun MM. The neural fate of consciously perceived and missed events in the attentional blink. Neuron. 2004;41(3):465-72.

71. Shulman GL, McAvoy MP, Cowan MC, Astafiev SV, Tansy AP, d'Avossa G, et al. Quantitative analysis of attention and detection signals during visual search. J Neurophysiol. 2003;90(5):3384-97.

72. Todd JJ, Fougnie D, Marois R. Visual short-term memory load suppresses temporo-parietal junction activity and induces inattentional blindness. Psychol Sci. 2005;16(12):965-72.

73. Kanai R, Paffen CL, Hogendoorn H, Verstraten FA. Time dilation in dynamic visual display. J Vis. 2006;6(12):1421-30.

74. Hayashi MJ, Valli A, Carlson S. Numerical quantity affects time estimation in the suprasecond range. Neurosci Lett. 2013;543:7-11.

75. Xuan B, Zhang D, He S, Chen X. Larger stimuli are judged to last longer. J Vis. 2007;7(10):2 1-5.

76. Pariyadath V, Eagleman DM. Brief subjective durations contract with repetition. J Vis. 2008;8(16):11 1-6.

77. Johnston A, Arnold DH, Nishida S. Spatially localized distortions of event time. Curr Biol. 2006;16(5):472-9.

78. Boynton GM, Finney EM. Orientation-specific adaptation in human visual cortex. J Neurosci. 2003;23(25):8781-7.

79. Wittmann M. The inner sense of time: how the brain creates a representation of duration. Nat Rev Neurosci. 2013;14(3):217-23.

80. Casini L, Vidal F. The SMAs: Neural Substrate of the Temporal Accumulator? Front Integr Neurosci. 2011;5:35.

81. Jin DZ, Fujii N, Graybiel AM. Neural representation of time in cortico-basal ganglia circuits. Proc Natl Acad Sci U S A. 2009;106(45):19156-61.

82. Duysens J, Schaafsma SJ, Orban GA. Cortical off response tuning for stimulus duration. Vision Res. 1996;36(20):3243-51.

**Supporting Information**

**S1 Data. The original data underlying each figure in the manuscript and supporting information.**

**S1 Fig. Plots of the beta values in the right SMG for each combination of shapes during the shape task.** (A) Plots of the beta values under each set of shapes at the peak coordinates of the clusters in the right SMG (*x*, *y*, *z* = 58, −42, 30) that showed duration adaptation effects during the time task in experiment 1 (see Fig 2A). (B) Plots of the beta values under each set of shapes at the peak coordinates of the clusters in the right SMG (*x*, *y*, *z* = 62, −34, 32) that showed a duration adaptation effect during the time task in experiment 2 (see Fig 2C). Letters on the *x*-axes represent combinations of shapes for reference and test stimuli: square-square (SS), circle-circle (CC), square-circle (SC), and circle-square (CS). Error bars indicate standard errors of the mean. Please refer to S1 Data for the numerical values underlying these figures.

**S2 Fig. Shape adaptation effects during the shape task in experiment 1.** (A) Cluster showing shape adaptation is shown in axial (bottom left, *z* = −18) slices of the standard brain. Plots of the beta values for each set of shapes during the shape task (B) and for each set of stimulus durations during the time task (C) at the peak coordinates of the cluster in the right parahippocampal gyrus (*x*, *y*, *z* = 20, −22, −18). The right parahippocampal gyrus showed a significant adaptation to the repetition of the same shape but not to the repetition of the same duration. Color scale indicates *T*-values. Letters on the *x*-axis represent combinations of shapes for reference and test stimuli: square-square (SS), circle-circle (CC), square-circle (SC), and circle-square (CS). Error bars indicate standard errors of the mean. Please refer to S1 Data for the numerical values underlying (B) and (C).

**S3 Fig. Greater stimulus duration adaptation during the time task than shape adaptation during the shape task in experiments 1 and 2.** Clusters highlighted by the contrast (duration adaptation during time task > shape adaptation during shape task) are shown for experiments 1 (A) and 2 (B). The color scale indicates the *T*-values.

**S4 Fig. Overlap of clusters showing duration adaptation effects during the time task and the shape task.** Red clusters represent areas showing duration adaptation effects during the time task (corresponding to the results shown in Fig 2A and 2C), and blue areas represent those showing duration adaptation effects during the shape task (corresponding to the results shown in Fig 3A and 3C) in experiment 1 (A) and experiment 2 (B). Overlapped areas are colored magenta, as shown in the legend.

**S1 Table. Anatomical labels and statistical results of experiment 1.**

**S2 Table. Anatomical labels and statistical results of experiment 2.**
